# Optimizing Screw Speed and Barrel Temperature for Textural and Nutritional Improvement of Soy-Based High-Moisture Extrudates

**DOI:** 10.3390/foods13111748

**Published:** 2024-06-02

**Authors:** Gabriela Ribeiro, María-Ysabel Piñero, Florencia Parle, Belén Blanco, Laura Roman

**Affiliations:** 1 CARTIF Technology Centre, Boecillo, 47151 Valladolid, Spain; gabrieladaconceica.ribeiro@estudiantes.uva.es (G.R.); marpin@cartif.es (M.-Y.P.); belbla@cartif.es (B.B.); 2 Food Technology Area, Department of Agricultural and Forestry Engineering, University of Valladolid, Av. Madrid, 50, 34004 Palencia, Spain; florenciaagustina.parle22@estudiantes.uva.es

**Keywords:** high-moisture extrusion, fibrous structuring, meat analogue, protein texturization, plant-based

## Abstract

Soy remains the legume protein of excellence for plant-based meat alternatives due to its fiber-forming potential. In this study, protein-rich powders from soy protein isolate (SPI), concentrate (SPC), and their mixture (SPM) were thoroughly characterized for their proximate composition, nutritional quality, and physicochemical properties to understand their structuring behavior during high-moisture extrusion. SPI presented higher degrees of protein denaturation and aggregation, least gelation concentration and lower essential amino acid contents. Thus, an SPI:SPC combination (1:9 ratio, 70% protein) was extruded at three different screw speeds (300, 350, and 400 rpm) and two temperature profiles (120 and 140 °C maximum temperature). The effects of the processing parameters on the extrudates were evaluated for their appearance (fibrousness), texture (TPA, cutting force, and anisotropy), color, protein structure (FTIR), and trypsin inhibitors. Higher temperatures resulted in softer and darker extrudates, with increased visual and instrumental anisotropy. Increasing screw speeds led to softer and lighter extrudates, without a clear fibrousness effect. β-sheet structures decreased and intermolecular aggregates (A1) increased after extrusion, especially at 140 °C, together with the formation of intramolecular aggregates (A2). Extrusion also significantly decreased the amount of trypsin inhibitors (>90%). This study demonstrates that extrusion parameters need to be carefully selected to achieve meat analogs with optimal textural and nutritional characteristics.

## 1. Introduction

In recent years, there has been a notable increase in plant-based meat innovation, driven by the convergence of consumer demands, market growth, and sustainable future food supply [1]. The use of plant proteins in food formulations is emerging as a popular option to sustain the food system against environmental limitations [2]. However, consumers do not want to compromise on the typical texture and flavor of meat. In an attempt to meet consumer expectations, meat analogs are produced using plant proteins through sustainable technologies such as extrusion (low- and high-moisture processes), aiming to mimic the characteristic fibrous anisotropic structure of meat [3]. High-moisture extrusion is currently the most predominant industrial technology for producing whole-cut meat analogs from plant proteins, involving extrusion processing at moisture levels of 40–80% coupled with a prolonged cooling die for fibrous texture development [3,4,5]. Despite the high initial investment cost of the equipment needed for high-moisture extrusion processing, this technology offers significant advantages, including scalability and versatility, reduced energy consumption and cost-effectiveness, minimized waste production, and the superior quality of the texturized products [5,6]. The resulting extruded products aim to achieve a meat-like texture by using plant-based material with a fibrous and dense structure, strong elasticity, and a high moisture content. In fact, these extruded products have been shown to possess sensory descriptors similar to those found in different animal whole-cuts, including beef, pork and chicken, although some other descriptors still represent a challenge (i.e., drier and less fibrous sensory perceptions) [7].

Extrusion is a high-temperature, short-time processing technique, inducing various changes to the shape/texture, structure, and composition of the raw materials. During extrusion, the raw materials are exposed to the effects of temperature, shear force, and pressure, leading to changes such as denaturation, oxidation, degradation, reassociation, and aggregation of proteins, carbohydrates, and/or lipids, and to the interaction between molecules that alter their molecular conformation, forming new structural assemblies and textures [8]. During extrusion, proteins undergo denaturation, unfolding, and molecular realignment to form a fibrous anisotropic structure upon cooling due to the directional shear force in the cooling die [4]. However, the molecular and colloidal mechanism for these fibers’ formation is still unclear due to the great differences in the purity and characteristics of the raw materials and the complexity of the extrusion process itself [9]. Therefore, evaluating both raw material characteristics and extrusion conditions is crucial to understand product quality [10]. Extrusion parameters such as moisture, barrel temperature, and screw speed, as well as indirect response parameters like the specific mechanical energy (SME), torque, and melt temperature and pressure, are key points for controlling product quality [11]. In addition, the properties of legume proteins used as raw materials, influenced by factors such as protein composition, purity, conformation, and extraction methods, are essential parameters to consider [9,12]. These determine physicochemical properties like solubility, water affinity, viscosity and gel formation, greatly influencing the extrusion process responses and impacting the overall quality of the texturized proteins.

In recent years, high-moisture cooking has been used to produce meat analogs with a variety of different sources of plant proteins [9,12,13,14,15]. Nonetheless, soy protein is by far the most studied plant protein in extrusion due to its gelation and fiber-forming properties, more balanced amino acid profiles, and more neutral flavor and color [16]. There are several soy protein ingredients commercially available, including flours, protein concentrates, isolates, as well as textured and hydrolyzed proteins. Soy protein isolate (SPI), the most refined form of soy with a protein content exceeding 90%, is commercially produced via protein solubilization at high temperatures, salts, or pHs, followed by its isoelectric precipitation and drying to remove unwanted components and improve protein yield. However, this process can lead to extensive protein denaturation, resulting in significant changes in its physicochemical properties. As a cost-effective alternative with reduced processing requirements to SPI, soy protein concentrate (SPC) has been used as a main raw material in the production of meat analogs [17] as well as soy flour combinations with SPI at differing ratios [5]. Although SPI has proved to result in fibrous anisotropic textures [4], comparatively, SPC-based meat analogs are considered to result in enhanced textural attributes with improved fibrousness under similar extrusion or shear cell processing conditions compared to SPI-based formulations [18,19,20]. In this sense, Grabowska et al. [20] reported that anisotropy during shear cell processing is fostered by the presence of two separate phases, either proteins-proteins or proteins-macromolecule (i.e., polysaccharides), which may be thermodynamically incompatible, preventing transverse protein aggregation upon longitudinal arrangement in the cooling die and forming elongated fibers. Similarly, Sandoval Murillo et al. [21] also reported that anisotropy during the extrusion of peas forms as a result of the presence of protein fractions with different hydration properties, leading to a multiphasic system, even when using a simple single-protein concentrate. However, although less-refined protein ingredients are generally preferred from the sustainability and processing standpoint, they usually contain a higher content of antinutritional factors, such as trypsin inhibitors [22,23], that should not be disregarded when formulating animal-meat alternatives, especially if these possess digestive enzyme-inhibitory activities. Although the effect of extrusion on reducing trypsin inhibitors (TIs) has been addressed at low-moisture extrusion conditions [24,25,26], there are very limited number of studies determining TI reductions under high-moisture extrusion conditions.

Several studies have focused on evaluating the effect of high-moisture extrusion process parameters on either the textural attributes or protein structural characteristics of soy-based extrudates. Moisture’s influence has been widely studied and recognized as one of the most important factors affecting the textural properties and/or fibrousness of the final product [5,27,28]. Others like Fang et al. [19] have examined the effect of SME on the extrudates’ quality, indicating that an increased SME resulted in darker products with a higher tensile strength and hardness, but a lower texturization degree. Regarding the effect of extrusion temperature, diverse results have been reported. In this regard, Chen et al. [27] indicated that cooking temperature only had a significant effect on the tensile strength of SPI extrudates, while hardness and chewiness were not affected, and no clear effect was seen for the degree of texturization. Conversely, Lin et al. [28] reported that higher temperatures produced softer and less chewy products, only slightly changing protein solubility and with no results reported for the extrudates’ fibrousness. In a more recent study, Wittek et al. [4] indicated that increasing the extrusion temperature enhanced the alignment of the proteins, resulting in a more pronounced visual fibrousness of the SPI. However, Dekkers et al. [29] reported that temperature effect was hardly visible when processing a pure SPI, while it did influence the shear-induced fibrous formation of pectin-SPI blends at both macroscopic and microscopic levels. Regarding the effect of screw speed, fewer studies have addressed its effect on the textural and protein structural characteristics of soy-based high-moisture extrudates. Pietsch et al. [17] evaluated both the effect of screw speed and barrel temperature on an SPC, reporting that, at the studied conditions, anisotropy occurred with higher temperatures, and no changes in protein–protein interactions were observed between the tested conditions. What is more, these authors reported that at screw speeds higher than 180 rpm and temperatures above 100 °C, the visual anisotropic fibrous structure was disrupted by the appearance of porous structures. Despite all the above studies denoting the influence of extrusion parameters on the quality of high-moisture extrudates, there is a pressing need for holistic studies that provide a more comprehensive understanding of these parameters, not only assessing macrostructural and textural changes but also integrating insights from the protein structural level and their aggregation state in conjunction with their nutritional quality.

Therefore, to better understand the effect of extrusion processing parameters on the textural and nutritional quality of high-moisture extruded meat analogs, this study evaluated the macro- and microstructure (i.e., visual fibrousness), color, textural attributes (TPA, cutting force and anisotropy), and trypsin inhibitor contents of soy protein-based extrudates. Moreover, the changes in the secondary structure of the proteins (identified via FTIR) occurring upon the extrusion of the meat analogs were also evaluated. Specifically, the high-moisture extrudates were produced from a well-characterized mixture of a soy protein isolate and concentrate, which was extruded at two different barrel temperature profiles (a 120 and 140 °C maximum temperature) and three screw speeds (300, 400, and 450 rpm). Furthermore, to understand soy protein structuring during high-moisture extrusion conditions, the protein-rich powders from a soy protein isolate, concentrate, and their mixture were previously characterized for their proximate composition, nutritional quality and physicochemical properties.

## 2. Materials and Methods

### 2.1. Materials

A commercial soy protein isolate (SPI) (ProFam 974) and concentrate (SPC) (Arcon SM) were purchased from Archer Daniels Midland Company (ADM, Chicago, IL, USA). A mixture (SPM) of both SPI and SPC protein powders was prepared by mixing SPI and SPC at a 1:9 ratio, to obtain ~77% (*w*/*w*, dry basis) of protein content.

### 2.2. Methods

#### 2.2.1. Proximate Composition of the Protein Ingredients

The proximate composition of SPI, SPC, and SPM was determined following AOAC standard methods for moisture, ash, total dietary fiber (TDF), and fat measurements. Moisture content was determined according to the AOAC 934.01 method [30] by drying the samples in an oven set at 105 °C for 16 h. Ash content was determined following the gravimetric AOAC 923.03 method [31]. TDF was determined according to the AOAC 991.43 method [32]. Total fat content was measured using a Soxhlet extraction in accordance with the AOAC 920.39 method [33]. Protein was determined following the combustion method according to AACC 46-30.01 [34] with an automated DUMAS protein analyzer (Leco CHN-828, St. Joseph MI, USA), using a conversion factor of 6.25 to calculate protein content from nitrogen. Total carbohydrates were calculated via weight difference. All determinations were conducted in duplicate.

#### 2.2.2. Protein Molecular Profile (SDS-PAGE)

Protein molecular profile was obtained via sodium dodecyl sulphate gel electrophoresis (SDS-PAGE) of the samples under reducing conditions. SDS-PAGE analysis was conducted using a vertical gel electrophoresis device (Mini-PROTEAN^®^ TGX™ Precast Protein Gels Bio-Rad^®^) following the method of Laemmli [35]. The molecular weight of the samples was estimated with a standard marker (Dual Color Standard, Bio-Rad^®^, Hercules, CA, USA), ranging from 10–250 kDa.

Briefly, 5 μL of each protein ingredient (20 mg protein/mL of ultrapure water) was collected in an Eppendorf tube and mixed with 5 µL of Sample Buffer Solution (consisting of 190 μL of Laemmli Sample Buffer and 10 μL of Native Sample Buffer (Bio-Rad)). Then, the mix was heated to 100 °C for 10 min. After that, 1 μL was collected and loaded into the wells. The loading volume of the marker was 5 μL. The buffer solution was loaded into the electrophoretic chamber, and consisted of a premixed electrophoresis buffer solution (25 mM Tris, 192 mM glycine, 0.1% SDS, pH 8.3, Bio-Rad^®^) diluted with distilled water in a 1:10 ratio. The electrophoretic separation was conducted by applying 150 V for 50 min. The gels were stained by adding 50 mL of Bio-Safe Coomassie Brilliant Blue dye (Bio-Rad^®^) for 1 h under continuous mixing. Finally, the gels were rinsed several times with distilled water and were allowed to discolor for 30 min before capturing the gels. The gels were prepared in duplicate.

#### 2.2.3. Amino Acid Composition of the Protein Ingredients

To determine the amino acid content of the SPI, SPC and SPM powders, 50 mg (±0.2 mg) of sample was weighed in quartz vials for microwave hydrolysis. In the hydrolysis rotor vessel of the microwave, 30 mL of 6 N HCl containing 400 μL of 90% phenol were added. The phenol was added to avoid the degradation of the tryptophan under acidic conditions. Following this, 200 μL of the hydrolysis solution (6 N HCl containing 1.2% phenol) was added to each quartz vial containing the samples to be hydrolyzed, then the vials were sealed and inserted into the rotor vessel. An inert atmosphere was created inside the rotor vessel using nitrogen and the microwave digestion protocol was performed. The microwave digestion was programmed at 650 W and the cycle consisted of a 3 min ramp to 160 °C, a hold period of 10 min and a final cooling down period of 20 min. After microwave digestion, the sample was completely dried using a dry heater set at 60 °C using nitrogen as a carrier gas, dissolved in 1 mL of 0.1 N HCl, filtered through a 0.22 μm nylon membrane, and transferred to HPLC vials for analysis.

Amino acid composition was analyzed according to Guideline 98/64/EG (1998) of the European Union [36]. The amino acid content was analyzed using high-performance liquid chromatography (HPLC) equipment (Agilent 1200, Agilent Technologies, Santa Clara, CA, USA) equipped with a UV detector (G1314B) and a Zorbax Eclipse Plus AAA column (4.6 × 150 mm × 3.5 µm) (Agilent Technologies, CA, USA) placed in a thermostatted column compartment kept at 40 °C. The separation and quantification of amino acids was conducted as precolumn derivatization with an OPA (o-phthalaldehyde) and FMOC reagent (2.5 mg/mL of 9-fluorenylmethylchloroformate in acetonitrile). Separation was obtained at a flow rate of 2 mL/min with a flow gradient consisting of 1.9 min at 0% B followed by a 18.1 min time to raise eluent B to 53%. Then, the column was washed at 100% B, followed by equilibration at 0%B in a total analysis time of 30 min. Mobile phase A consisted of 40 mM of NaH_2_PO_4_ (pH 7.8), while mobile phase B was a mix of acetonitrile: methanol: water (45:45:10, *v*/*v*). Calibration curves were obtained for each amino acid of interest by using different concentrations of a standard amino acid mix (Amino Acid Standard, AAS18, Sigma Aldrich, San Luis, MO, USA). Protein hydrolysis and HPLC analyses were conducted in duplicates. The amount of each individual amino acid was expressed in g/100 g of protein and the content of the essential amino acids was compared to the FAO/WHO (2013) reference pattern of essential amino acids requirements for adults. The ratio of essential to total amino acids was reported as E/T (%).

#### 2.2.4. Thermal Properties

A differential scanning calorimeter Q-20 (TA instruments, Crawley, UK) equipped with a refrigerated cooling system (RCS 40) was used to evaluate the native state of the protein ingredients. The calibration for enthalpy and temperature was completed using indium. Tzero aluminum hermetic DSC pans (TA-Instruments, New Castle, DE, USA) were employed. An empty pan was used as a reference and dry nitrogen at a flow rate of 50 mL/min was used as a purge gas. For DSC analysis, protein powders and ultra-pure water were mixed in a 1:3 ratio (*w*/*w*) and hermetically sealed. Samples were kept at 25 °C for 24 h and heated from 30 to 120 °C at 5 °C/min. The enthalpy of protein denaturation (ΔH_d_ in J/g protein) and the temperature of denaturation at the peak maximum (T_d_) were reported. Three replicates per sample were conducted.

#### 2.2.5. Physicochemical Properties

##### Water-Binding and Oil-Binding Capacity

The water-binding capacity (WBC) measurement was carried out following the method AACC 56.30.01 [37] with small modifications. An amount of 1 g of protein powder was transferred into a centrifuge tube and 5 g of distilled water was added. After vortex mixing for 1 min, the sample was centrifuged for 10 min at 2000× *g* and unbound water was discarded. Results were reported as the weight of the water retained per 1 g of sample (g/g).

The oil binding capacity (OBC) measurement was carried out following the procedure used by Lin et al. [38], with slight modifications; 0.5 g of protein powder was transferred into a centrifuge tube and mixed with 3 mL of sunflower oil. Samples were vortex-mixed for 1 min, followed by centrifugation for 25 min at 1610× *g*. The unbound oil after centrifugation was discarded with the help of a pipette. The density of the sunflower oil was used to express the result in mL of the oil bound per g of sample. Duplicate measurements were performed for WBC and OBC of each sample type.

##### Least Gelation Concentration

The least gelation concentration (LGC) was determined as reported by Adebiyi and Aluko [39]. Briefly, 5 mL dispersions of SPI, SPC, and SPM were prepared at concentrations ranging from 6 to 20% (*w*/*v*) of protein powder in distilled water. Subsequently, samples were vortex-mixed and the mixtures were heated for 1 h at 95 °C in a water bath (BOE-4 series, Raypa, Barcelona, Spain), cooled down rapidly under tap water, and kept at 4 °C for 2 h. After cooling, the tubes were inverted and gel formation was visually determined at each concentration. The LGC was expressed as the lowest concentration at which the sample did not slip or fall down the side of the test tubes. LGC analyses were performed in duplicates.

##### Foaming and Emulsifying Properties

Foaming capacity (FC) and foaming stability (FS) were determined following the procedure described by Lin et al. [38] with some modifications. Briefly, 3 g of protein powder was suspended in 100 mL of distilled water. The mixture was homogenized with a T18 homogenizer (IKA, Staufen, Germany) at a speed of 10.000 rpm for 2 min and subsequently transferred to a graduated cylinder. Then, the total volume and the foam volume (1 min after homogenization) were recorded. Measurements of the total and foam volumes were taken at 1 min (the initial foam volume), 30 min and 120 min intervals. Foaming capacity was expressed as the relative percentage of the initial foam volume (after 1 min) to the total volume of the sample. Foam stability was defined as the relative percentage of the foam volume remaining after 120 min to the initial foam volume.

The emulsifying activity index (EAI) and emulsion stability index (ESI) were determined following the method described by Pearce and Kinsella [40] with small modifications. An amount of 6 mL of a 0.5% (*w*/*w*) protein solution and 2 mL of sunflower oil were homogenized for 2 min at 10.000 rpm using a T18 homogenizer (IKA, Staufen, Germany). A 0.1% solution of SDS was prepared and used as a blank. Subsequently, 50 μL of the homogenized emulsion sample was diluted in 5 mL of SDS solution (0.1% *m*/*v*) and mixed using a vortex mixer. The absorbance of the mixture was measured at 500 nm using a UV–visible spectrophotometer (UV-1603, Shimadzu, Kyoto, Japan), using plastic cuvettes (1 cm path length). Measurements were taken immediately after sample preparation and after 10 min. The EAI and ESI were calculated according to the equations reported in Karaca et al. [41]:

EAI (m^2^/g) = (2 × 2.303 × A_0_ × N)/(c × ø × 10,000)

ESI (min) = A_0_/(A_0_ − A_10_) × 10

where, 2 and 2.303 are constant values [40], A_0_ is the absorbance of the diluted emulsion immediately after homogenization, N is the dilution factor (×100), c is the weight of the protein per volume (g/mL), ø is the oil volume fraction of the emulsion, A_10_ is the absorbance of the diluted emulsion after 10 min. Duplicate measurements were performed for the FC/FS and the EAI/ESI for each sample.

##### Pasting Properties

The viscosity properties of SPI, SPC and SPM protein powders under a heating–holding–cooling cycle were analyzed following the standard 95 °C method (RVA Method 50.01 reported by Perkin Elmer (Waltham, MA, USA) [42] using a Rapid Visco Analyzer (RVA-4800, Perten Instruments, Sydney, Australia). For the three protein powders subjected to the standard 95 °C method, samples were first maintained at 50 °C under a shear rate at 960 rpm for 10 s for adequate mixing, and after that the speed was maintained at 160 rpm for the rest of the test. Subsequently, the slurry was held at 50 °C for 60 s and then heated to 95 °C with a temperature increase of 6 °C/min, held at 95 °C for 5 min, and finally cooled to 50 °C at 6 °C/min, and kept at 50 °C for 2 min. Furthermore, the viscosity profile of the mixed protein powder (SPM), used for further extrusion experiments, was obtained at three different maximum hold temperatures, following the standard pasting cycle at 95 °C, as described above, as well as the high-temperature pasting cycle at 120 °C and 140 °C, described in the RVA Method 50.01-Pulse Flour and Pulse Starch Method [42]. The parameters used in the three pasting cycles at a maximum heating temperature of 95, 120, and 140 °C are reported in Table 1. For all the experiments, samples were prepared by mixing the protein powders (4.5 g) with distilled water (24 g) at a 14% moisture basis. From the viscosity profiles, the maximum peak viscosity (PV) and final peak viscosity were recorded (FV) and included in the Supplementary Materials (Table S1). The viscosity tests were performed in duplicates.

#### 2.2.6. Extrusion Processing

Extrusion experiments were conducted on a semi-industrial Evolum 25 corotating twin-screw extruder (CLEXTRAL, Firminy, France), able to work at a maximum solid feeding of 25 kg/h and a maximum liquid feeding of 40 l/h via volumetric dosing. The screw diameter (D) was 25 mm, and the screw length (L) was 600 mm, resulting in a length/diameter (L/D) ratio of 24:1. The barrel consisted of six independent temperature-controlled zones (Z). A LWFD5-20 volumetric feeder (K-Tron Corp., Pitman, NJ, USA) was used to feed the dry protein powder into the barrel. The feeder was calibrated and adjusted beforehand. Water was injected directly into the feeding zone, in the second barrel section, using a Super K pump (DKM-CLEXTRAL, Firminy, France) to maintain the target moisture content. The process conditions were monitored from a control panel (see Table 2). A thermoregulated cooling die (450 × 30 × 4, L × W × H) with an internal rectangular section of 30 mm × 4 mm was attached to the end of the extruder. The temperature of the cooling die was kept at 80 °C.

An experimental design was used to investigate the effect of screw speed and extrusion temperature on the quality of the high-moisture extruded meat analogs. The extrusion conditions are summarized in Table 2. A moisture content of 69% was selected and kept constant throughout all the experiments as this moisture content resulted in the most fibrous appearance in previous trials. The solid feed rate and water flow rate were 2.8 kg/h and 5.1 l/h, respectively. The screw speed was set to 300, 350, and 400 rpm, resulting in low (LS)-, medium (MS)-, and high (HS)-speed conditions, respectively. Furthermore, two different temperatures profiles were used. The extruder temperatures in the six-barrel zones (from the first barrel to the end plate) were set at 30, 50, 75, 105, 115, 120 °C (low temperature processing, LT) and 30, 70, 95, 115, 125, and 140 °C (high temperature processing, HT) to achieve two different thermal energies during extrusion.

Furthermore, the extruder responses including the motor torque, the melt pressure and melt temperature at the extruder exit, and Specific Mechanical energy (SME) were recorded online during the trials. The specific mechanical energy (SME; kJ/kg), defined as the amount of work supplied from the driving motor into the material being extruded, was used to quantify the severity of the shear forces during the extrusion process. The SME was calculated according to the following equation:

SME (kJ/kg) = 2π × n × T/MFR

where n is the screw speed (rpm), T is the torque (Nm), and MFR is the mass flow rate (kg/h).

The samples were collected after the stabilization of the operating conditions for each set of parameters. The protein extrudates were cut into rectangular strips as they exited the cooling die. The samples were allowed to cool down, stored in sealed plastic bags and kept at −18 °C until further analysis was conducted. The moisture of the extruded products was determined after thawing, before textural and protein structural analyses, according to the AOAC 934.01 method [30].

#### 2.2.7. Evaluation of the Visual, Textural, Nutritional, and Protein Structural Changes upon Extrusion

##### Macro- and Microscopical Evaluation

The macrostructure of the samples was evaluated using a phone camera. A piece of each extruded sample was manually broken into halves parallel to the flow direction to observe the fibrous anisotropic formation. The microstructure analysis of the extrudates was conducted using a scanning electron microscope (SEM) (Quanta 200, FEI, Eindhoven, The Netherlands). Frozen, extruded samples were thawed 4 h before analysis at room temperature, cut transversal to the flow direction, and placed on double-sided adhesive. Images of the samples were taken with the SEM at different magnifications, in low vacuum conditions, and with an accelerating voltage of 12.50 kV.

##### Color Evaluation

The color attributes of the protein powders and the produced extrudates were measured using a spectrophotometer (CM-3500d, Konica Minolta, Osaka, Japan). Calibration was performed with a zero calibration box and a white calibration plate (CM-A124; CM-A120, Konica Minolta, Osaka, Japan) before the measurements. Samples were measured using a Petri dish CM-A128, and results were obtained using a D65 standard illuminator and 2° observer. The color of the extrudates and protein powders was expressed in the CIELab color space, as parameters of L* (lightness, 0 = black and 100 = white), a* (−a/+a, greenness or redness), and b* (−b/+b, blueness or yellowness). Four measurements were taken at random locations on the surface of each sample. The total color differences (Δ*E*) of the extrudates were calculated using the following equation:

$$\Delta E=\sqrt{{\left(\Delta L*\right)}^{2}+{\left(\Delta a*\right)}^{2}+{\left(\Delta b*\right)}^{2}}$$

where, ΔL*, Δa*, and Δb* are the differences between the extrudates and the SPM, the protein powder used for the extrusion experiments.

##### Protein Secondary Structure

The secondary structure of the native and extruded proteins was studied using Fourier-Transform Infrared Spectroscopy (FTIR) by analyzing the amide I band (the stretching vibrations of C=O in the peptide bond) using a Nicolet IS50 FTIR spectrometer (Thermo Scientific, Waltham, MA, USA) coupled to an attenuated total reflectance (ATR) cell. The spectrum was taken by averaging 64 scans at a 4 cm^−1^ resolution in the wavenumber range of 600 to 4000 cm^−1^. A blank was performed before each spectrum collection. Extruded proteins were freeze-dried and milled before FTIR analysis to remove water interference from the amide I region. A Gaussian/Lorentzian deconvolution procedure of the Amide I band (1600–1700 cm^−1^) was conducted using OMNIC FTIR software (version 9.2.86, ThermoFisher Scientific, USA), enabling the identification of various secondary structures, including intermolecular aggregated structures (A1~range 1612–1620 cm^−1^), parallel β-sheets (range 1630–1638 cm^−1^), random coils and α-helices (range 1640–1660 cm^−1^), β-turns (range 1660–1680 cm^−1^), antiparallel β-sheets (range 1680–1688 cm^−1^) and intramolecular aggregated structures (A2~range 1690–1695 cm^−1^) [43,44,45]. As reported by Fevzioglu et al. [46], an overlapping of random coil and α-helix peak structures occurs in the range of 1640–1660 cm−^1^. Given that correctly assigning these peaks has been described as challenging [47], in the present study, peaks in that range were considered as α-helices. The relative proportions of the identified structures were calculated by integrating their respective deconvoluted bands and dividing them by the sum of the areas of all identified structures. Examples of the deconvoluted amide I band are reported in the Supplementary Materials (Figure S1). Baseline correction and normalization was performed before deconvolution. FTIR analyses were performed at least in triplicate.

##### Textural Properties

The textural properties of the extruded samples were evaluated by texture profile analysis (TPA) and a cutting test using a TA.XTPlusC 650 texture analyzer (Stable Micro-Systems, Godalming, UK). All samples were thawed at 25 °C and cut into a square shape of 20 mm × 20 mm before analysis. For TPA analyses, samples were subjected to a double compression test using a P/36 probe to compress 50% of its original thickness at a speed of 1 mm/s. The textural properties for hardness (N), springiness, resilience and cohesiveness were then recorded [27].

For cutting test evaluation, the sample was cut using a Meullenet Owens Razor Shear Blade (A/MORS) (Stable Micro-Systems, UK) to 75% of its original thickness at a speed of 2 mm/s [27,48]. Samples were evaluated parallel (with longitudinal cutting force, FL) and perpendicular (with transversal cutting force, FT) to the flow direction of the extrudates from the cooling die. The maximum cutting strength was calculated using the maximum force (N) obtained from Exponent connect software version 8.7.07 (Stable Micro-Systems, Godalming, UK) at the maximum fracture point per the flow direction. The maximum cutting strength at the FL and FT and the anisotropic index, defined as the ratio of the maximum cutting force between the FL and FT, was used to evaluate the fibrous formation. Equal values (FL = FT) indicate uniform texture with low material anisotropy. All textural determinations were performed with at least five replicates.

##### Antinutritional Factors (Trypsin Inhibitor Activity)

The quantitative evaluation of trypsin inhibitor activity in fresh native and extruded samples was performed in duplicates via a spectrophotometric method according to the standardized ISO method 14902 [49]. Briefly, 1 g of sample was suspended in 50 mL of 0.01 M NaOH, and the pH was adjusted to 9.4–9.6 with HCl, stored and further diluted with distilled water. A 1 mL sample was withdrawn and properly diluted to achieve 40–60% inhibition of the trypsin enzymatic activity. For the enzymatic reaction, a standard solution of trypsin was added to a mixture containing the properly diluted sample solution and a solution of α-N-benzoyl-l-arginine-4-nitroanilide hydrochloride (BAPA) and incubated at 37 °C for 10 min. After incubation, the reaction was quenched with the addition of 30% acetic acid. Blank reactions were simultaneously carried out. The reaction mixtures were then centrifuged, and their absorbance was measured at 410 nm, calculating the trypsin inhibitor activity (TIA) as the mg of the inhibited pure trypsin per g of sample.

### 2.3. Statistical Analysis

Statistical analysis from at least two independent measurements was conducted using Statgraphics Centurion 19-X64 software (Statgraphics Technologies, Inc., Warrenton, VA, USA). Data were expressed as mean ± standard deviation (SD). Significant differences between the parameters for the different samples were studied via a one-way analysis of variance (ANOVA). Fisher’s least significant difference (LSD) was used to describe means with 95% confidence intervals.

## 3. Results and Discussion

### 3.1. Proximate Composition of Protein Powders

The proximate composition, amino acid and protein composition, as well as the physicochemical properties of the protein powders are key parameters in the selection of raw materials for high-moisture extrusion processing, as these will determine the behavior of the protein-rich matrix during thermo-mechanical processing and, in turn, the characteristics of the final product.

The proximate compositions of the soy protein isolate (SPI) and concentrate (SPC), used to prepare the protein mixture (SPM) for extrusion experiments, are listed in Table 3. The moisture content ranged between 6 and 8% for all protein powders. The protein content was significantly higher for SPI (95% protein), compared to SPC (74% protein). The soy protein mix, SPM (SPI:SPC 1:9, *w*/*w*), had a similar protein content to SPC as SPC was the major component in the mixture. Regarding the fiber contents, while it was negligible in SPI (<1%), up to 4% of the fiber was found in SPC. This fiber fraction would mainly consist of cellulose, hemicellulose and pectin polysaccharides. Furthermore, in SPC and, hence, SPM, an important amount of other carbohydrates was present, which may correspond to small sugars, starch or oligosaccharides not accounted for in the crude fiber fraction [50]. The oil content was very low (<1%) in all samples denoting a high efficiency in fat removal from the oilseed during oil extraction and further protein extraction and purification. Similar to fat content, no significant differences were found for ash content (<5%).

### 3.2. Protein Molecular Properties

**Figure 1:**
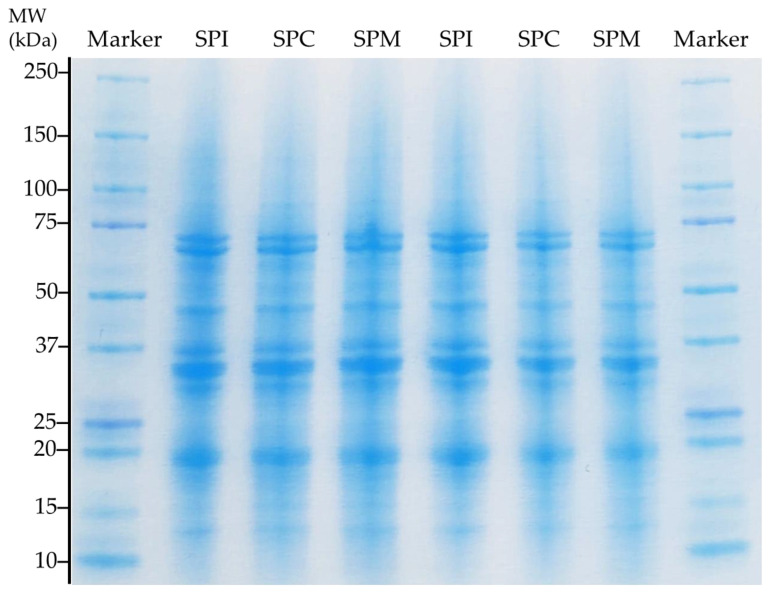
Gel electrophoresis profile at disassociating (SDS-PAGE) and reducing conditions for the soy protein isolate (SPI), concentrate (SPC) and their mixture (SPM) performed in duplicate.

To identify differences in the protein compositions between the SPI and SPC, the gel electrophoresis (SDS-PAGE) under reducing conditions (Figure 1) and the amino acid composition (Table 4) of the powders was evaluated. Electrophoretic profiles denoted that the soybean protein powders were mainly composed of subunits from their glycinin (11S) and β-conglycinin (7S) protein fractions. The 11S/7S ratio has been found to affect the degree of texturization of the soy protein [51]. In this sense, the three protein powders exhibited similar protein profiles with bands of different intensities between ~15 and 75 KDa. The three intense bands between 75 and 50 KDa were associated with the subunits of the trimeric β-conglycinin, α’ (~75 KDa), α (~70 KDa) and β (~50 KDa) and the other two bands corresponded to the acidic (~40–32 KDa) and basic (~20 KDa) subunits of glycinin. Similar bands were reported by Liu et al. [52], Flory and Alavi [53], and Xia et al. [54] in soy protein concentrates and isolates. In addition to the bands from the protein fractions mentioned above, bands of lower intensities were observed, including faint low-molecular-weight bands between 10 and 15 kDa. These bands could correspond to albumins and/or other low-molecular-weight proteins [9].

The amino acid compositions of the protein powders are shown in Table 4. The amino acid compositions were similar among the protein powders, denoting no significant differences between the SPI, SPC and SPM for histidine, valine, methionine, phenylalanine, isoleucine, leucine, tryptophan, glycine, arginine, tyrosine, cysteine, aspartic acid, glutamic acid, serine, and alanine. All samples showed high aspartic acid and glutamic acid contents, and a low content of methionine, tryptophan, and cysteine. Similar results were reported for the SPI by Fernández-Quintela et al. [55] and Mateen and Singh [56]. Regarding the proportion of essential to non-essential amino acids (E/NE), a decrease in the E/NE ratio was observed in SPI as compared to SPC, due to the lower presence of the essential amino acid threonine and lysine in the SPI, which could be attributed to factors such as the extraction process and the initial raw material composition [57]. As for the daily adult intake requirements recommended by the FAO, soy protein powders met or exceeded the patterns for amino acid requirements for all the essential amino acids except for methionine [56,58].

**Table 4:** Amino acid profile of protein powders expressed in g of amino acid per 100 g of protein and the daily requirement of essential amino acids.

Amino Acid	SPI	SPC	SPM	Daily Requirement **
Histidine *	2.83 ± 0.28 a	3.02 ± 0.25 a	2.91 ± 0.25 a	1.50
Threonine *	2.97 ± 0.23 a	3.24 ± 0.25 b	3.11 ± 0.25 a	2.30
Valine *	3.12 ± 0.42 a	3.26 ± 0.46 a	3.19 ± 0.47 a	3.90
Methionine *	1.33 ± 0.21 a	1.36 ± 0.22 a	1.38 ± 0.22 a	2.20 ***
Phenylalanine *	4.18 ± 0.24 a	4.14 ± 0.25 a	4.05 ± 0.25 a	4.00
Isoleucine *	2.94 ± 0.30 a	2.97 ± 0.33 a	2.89 ± 0.33 a	3.00
Leucine *	6.60 ± 1.02 a	6.75 ± 1.75 a	6.57± 1.26 a	5.90
Lysine *	6.22 ± 0.14 a	6.51 ± 0.11 b	6.37 ± 0.11 ab	4.50
Tryptophan *	0.15 ±0.70 a	0.12 ±0.86 a	0.11 ±0.94 a	0.60
Glycine	3.69 ± 0.35 a	3.89 ± 0.38 a	3.77 ± 0.38 a	
Arginine	5.87 ±1.27 a	5.49 ± 0.92 a	5.49 ± 0.95 a	
Tyrosine	3.15 ± 0.8 a	2.99 ± 0.7 a	2.91 ± 0.8 a	
Cysteine	0.88 ± 0.73 a	1.00 ± 0.67 a	0.98 ± 0.70 a	
Proline	4.87 ± 0.56 a	3.56 ± 0.51 b	4.00 ± 0.25 ab	
Aspartic acid	10.57 ± 1.69 a	10.59 ± 1.81 a	10.31 ± 1.76 a	
Glutamic acid	16.93 ± 1.81 a	16.46 ± 1.92 a	16.16 ± 1.94 a	
Serine	4.56 ± 0.43 a	4.69 ± 0.75 a	4.56 ± 0.55 a	
Alanine	3.83 ± 0.40 a	4.22 ± 0.42 a	4.04 ± 0.43 a	
E/T (%)	30.32	31.35	30.58	
E/NE (%)	57.44	59.93	58.54	

Mean ± standard deviation followed by different letters within each row indicate significant differences (*p* < 0.05). SPI: soy protein isolate, SPC: soy protein concentrate, SPM: soy protein mix. E/T = the proportion of essential amino acids to total amino acids; E/NE = the proportion of essential amino acids to non-essential amino acids. * Essential amino acid. ** Values of the daily requirement corresponding to the adult (>18 y) maintenance amino acid pattern (g/100 g protein) recommended in the dietary protein quality evaluation in the human nutrition report (FAO, 2013) [59]. *** Daily requirement for sulfur-containing amino acids (Methionine + Cysteine = 2.2 g/100 g).

Differential scanning calorimetry (DSC) was used to study the native state of the protein ingredients (Table 3). Calorimetric results indicated extensive protein denaturation in the SPI as no enthalpic transitions were observed in this sample, suggesting important bonds in the creation and maintenance of the protein structure were disrupted during its purification. Nonetheless, the SPC and SPM still exhibited two endothermic peaks in the DSC thermogram. This would align with the results observed for the secondary structure components of the different unextruded soy proteins (see Section 3.4.2), with higher intermolecular protein aggregated structures (A1~1617–1624 cm^−1^) observed for the SPI as compared with its SPC counterpart. The two endothermic peaks found in the SPC and SPM were attributed to the main protein components of the soybean protein, β-conglycinin (7S) and glycinin (11S), the main protein fractions identified in the SDS-PAGE profiles. The transition temperature of the peak corresponding to 7S was observed at ~86 °C for the SPC and the SPM. This peak exhibited significantly lower enthalpy (1.4–1.8 J/g protein) than the peak attributed to 11S, in agreement with previous observations [44,60]. The denaturation temperature of 11S was much higher than that of 7S, with a T_d_ at ~116 °C and an enthalpy of 3.2–3.4 J/g, which was similar between the SPC and the SPM. Temperatures of denaturation were in the range of those reported in the literature for 7S (78–90 °C) and 11S (95–109 °C) [44,60,61,62] with variations attributed to the soybean characteristics, such as processing conditions or salt contents [60,63], or the moisture contents during DSC analyses [64], among others.

### 3.3. Physicochemical Properties of Protein Powders

The physicochemical properties of protein powders were evaluated in terms of their water- and oil-binding capacity, gel-forming ability and viscosity, and foaming and emulsifying properties (Table 3). Regarding the WBC, no significant differences were observed between SPC and SPM. Both protein powders showed a higher WBC than SPI, which can be attributed to the higher nativity state of the SPC protein (fewer exposed hydrophobic patches) [65] and/or the higher amount of non-protein components, especially high-molecular fibers, with high water-binding affinities [66]. The OBC of proteins is partly related to their intrinsic hydrophobicity, as non-polar amino acids can interact with aliphatic chains of lipids, with extended surface hydrophobicity also fostering protein aggregation rather than protein–lipid interaction [65]. However, no significant differences (*p* < 0.05) were observed between the protein powders.

The foaming and emulsifying properties were evaluated as an indicator of the surfactant properties of the protein ingredients. These properties depend on intrinsic factors such as protein structure and composition, their colloidal properties [67], as well as the presence of some other components, such as carbohydrates [68]. In this regard, conglycinin has better surfactant properties than glycinin due to its smaller molecular mass, higher structural flexibility (being easier to unfold), the presence of glycosylated groups and its higher hydrophobicity [69]. SPC exhibited a higher initial foaming capacity than SPI, while the latter was characterized by a greater foam stability, which could be related to their different nativity state, as native plant proteins have limited foaming properties due to their compact structure [67]. In addition, SPI presented both a higher emulsion activity and stability. Meanwhile, no significant differences (*p* < 0.05) were observed between SPC and SPM. The better emulsifying properties of SPI could be related to its higher effective protein concentration or extended denaturation rather than differences in the 7S/11S ratio between SPC and SPI. In this sense, pulse protein concentrates have been reported to possess lower emulsifying activity than soy protein isolates [70,71].

Globular proteins are able to form gels when they are heat-denatured in aqueous solutions. The least gelation concentration (LGC) indicates the protein ability to create a protein network that can entrap water upon heating and subsequent cooling, forming a heat-set gel [12]. Therefore, a lower LGC indicates that the protein has a better capacity to form gels. Despite its lower protein content, SPI showed a higher LGC after heating than the SPM and especially SPC, with LGC values ranging from 12–14% solids, similar to those found by Flory and Alavi [53] for a commercial SPI and SPC (14%). The lower LGC of the SPI could be related to the lower native and greater aggregation states of the denatured SPI, whose extensive aggregation would result in a higher protein concentration needed to form the network [12]. Therefore, limited thermal denaturation during processing may have a beneficial effect on the functionality of the protein ingredients, especially if further heat processing is not performed at temperatures high enough to disrupt the aggregates.

**Figure 2:**
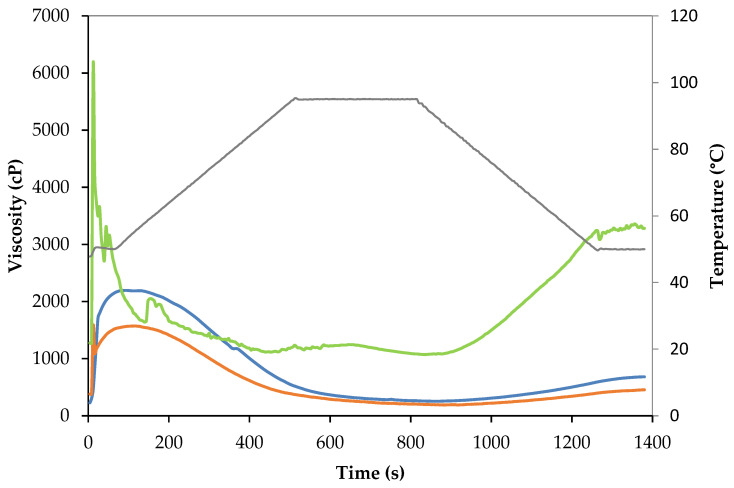
Viscosity profile of the protein powders. The green line is pasting profile of the soy protein isolate; the red line is the pasting profile of the soy protein concentrate; the blue line is pasting profile of the soy protein mix; the grey line represents the temperature profile during the viscosity test.

To further evaluate the gelation potential of the protein ingredients, their viscosity profiles under a heating–holding–cooling cycle were evaluated under low shear stirring conditions. The viscosity profile was evaluated at a 16% solids concentration, and, hence, was above the LGC of all protein ingredients. When protein powders were heated up to 95 °C (Figure 2), all protein powders exhibited cold-swelling properties, which further solubilized upon heating, decreasing their viscosity, which was later increased upon the final cooling. Flory and Alavi [53] obtained similar results for the viscosity profile of an SPI and SPC. These authors classified protein ingredients based on the peak viscosity development during the heating cycle, those proteins with peak viscosity before 4 min were categorized as having cold-swelling properties while peaks later than 4 min were indicative of heat-swelling properties. In the heating–cooling cycle, the SPI was characterized by a high initial viscosity that decreased with the temperature [72]. SPI showed a higher initial swelling, a faster solubilization and a higher final viscosity than SPC or SPM. SPI was characterized as having cold-swelling proteins, what allows it to easily form gels [53]. When soy proteins unfold due to shearing or heat and are exposed to water, their non-polar and sulfhydryl groups readily form hydrophobic and disulfide bonds that cause the proteins to aggregate and produce an increase in viscosity [73]. In the case of SPC, the formed network was weaker (i.e., at a lower final viscosity) which could be attributed to the presence of other components such as carbohydrates that interfere with protein aggregation and the formation of the protein network as well as with its lower protein content (see Table 3). Mixing the two protein powders (the SPI and the SPC) had a synergetic effect on the viscosity profile of the SPM, showing a viscosity profile similar to that of the SPC, but with enhanced viscosity.

**Figure 3:**
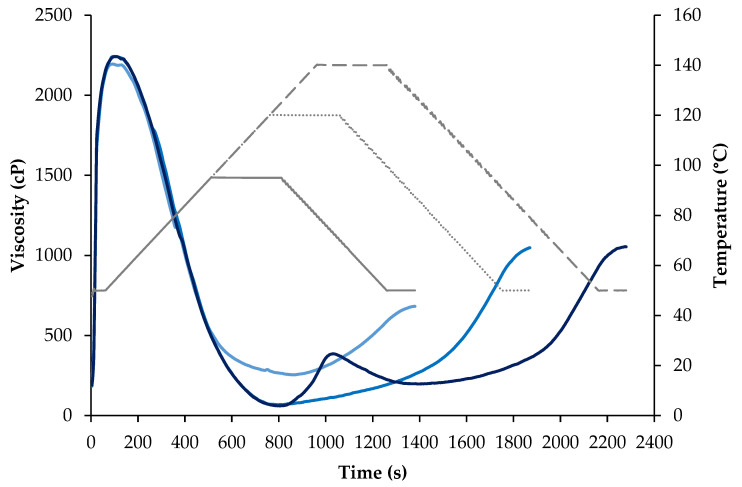
Viscosity profile of the soy protein powder mix, SPM, at three different maximum temperature profiles (95, 120, and 140 °C). The light blue line indicates the pasting profile with a maximum holding temperature at 95 °C; the medium blue line is the pasting profile with a maximum holding temperature of 120 °C; the dark blue line is the pasting profile with a maximum holding temperature of 140 °C, and the continuous, dotted, and line-broken grey lines represent the temperature profiles for the 95, 120, and 140 °C viscosity cycles, respectively.

Viscosity plays a crucial role in altering the flow behavior and the mechanical energy input in extrusion cooking. For this reason, the viscosity profiles of the SPM powder used for the extrusion process were further determined at three different maximum temperatures; in addition to 95 °C, profiles were also determined at 120 °C and 140 °C, which were the maximum barrel temperatures reached during the extrusion process at low (LT)- and high (HT)- temperature conditions (120 and 140 °C), respectively. Figure 3 shows the three viscosity profiles at 95, 120 and 140 °C maximum heating temperatures. No significant differences (*p* < 0.05) were observed in the initial cold-peak viscosity as cooking was performed at the same heating rate. Nonetheless, after the cold-peak viscosity, a higher drop in viscosity (i.e., the minimum viscosity) was obtained with the temperature profile of 120 and 140 °C, during the maximum heating temperature compared with the profile at 95 °C, with even the appearance of a second peak at 140 °C conditions. The sudden increase in viscosity at a 140 °C holding temperature could be explained partly by undenatured protein fractions or the disruption of protein aggregates that may not be disrupted at milder heating conditions, based on the higher thermal stability of glycinin observed in DSC results. Interestingly, a second peak during heating has also been observed in a hemp protein concentrate processed at 130 °C [72] which possesses a predominance of the 11S edestin fraction, similar to soy glycinin. Upon cooling, viscosity results showed that as the maximum cooking temperature increased, SPM exhibited a greater capacity to form a structured gel, increasing its final consistency, which may be the result of an increased extent of protein denaturation at elevated temperatures (glycinin) and/or a structure breakdown with the rupture of aggregates. Thus, as the temperature increased, the proteins denatured and interacted with water; and the unfolded proteins led to stronger interactions, resulting in greater water retention and a higher capacity to form a structured gel.

### 3.4. Extrusion Processing of Soy Protein Powders

Extrusion experiments to produce high-moisture extrudates were carried out using the SPM as the raw material, which was produced by mixing the SPC and the SPI. The use of less refined ingredients has been reported to be beneficial for allowing phase separation and a better fibrousness of high-moisture extruded meat analogs [20]. During extrusion processing, the effect of two different processing parameters, barrel temperatures and screw speed, were analyzed (see Table 2). Extrusion tests were carried out using two different barrel temperature profiles, low (LT) at 120 °C, and high (HT) at a 140 °C maximum temperature, that resulted in product temperatures of 115 and 130 °C, respectively. Furthermore, three different speeds were used, low, medium, and high, at 300 (LS), 350 (MS), and 400 (HS) rpm, respectively. This resulted in six different extrudates, LT-LS, LT-MS, LT-HS, HT-LS, HT-MS, and HT-HS. The influence of these process parameters on the visual anisotropy, textural and color attributes as well as the antinutritional factors of the extruded meat analogs were evaluated, together with the changes in the protein secondary structure occurring upon extrusion.

#### 3.4.1. Macro- and Microscopic Evaluation of Extruded Meat Analogs

**Figure 4:**
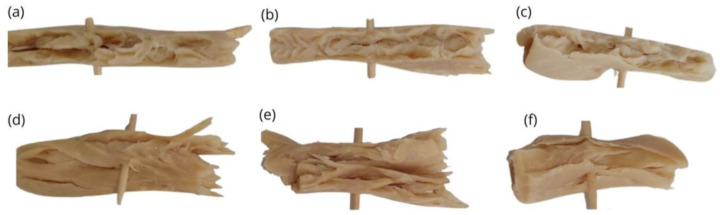
Visual appearance of the high-moisture extruded meat analogs cut longitudinally to the flow direction under different temperatures and screw speed conditions. (**a**) LT-LS; (**b**) LT-MS; (**c**) LT-HS; (**d**) HT-LS; (**e**) HT-MS; (**f**) HT-HS. LT: low temperature; HT: high temperature; LS: low speed; MS: medium speed; HS: high speed.

Figure 4 shows the macrostructural appearance of the extrudates obtained at different process conditions. All extruded meat analogs had visible anisotropy in the form of fibrous or layered structures, with predominantly lengthwise-oriented fibers upon tearing [48]. It is worth noting that SPM contained significant amounts of carbohydrates (>10%), especially fibers (see Table 1), which would have facilitated the phase separation of protein domains and the formation of layered structures upon cooling [9,74]. As two or more thermodynamically incompatible phases can prevent a transverse aggregation of the proteins and benefit the longitudinal arrangement forming the fibers [20]. As for the processing parameters, more lengthwise-oriented fibers were formed after extrusion at a higher barrel temperature, in agreement with Osen et al. [48]. Higher temperatures have been reported to promote more disassembled protein subunits, which could realign more efficiently via the directional shear force inside the cooling die creating more elongated fibers [75], something that was also suggested when evaluating the viscosity profile of SPM at 120 and 140 °C (see Figure 3). Furthermore, at the same temperature conditions, smaller differences in fibrousness could be observed according to the screw speed.

**Figure 5:**
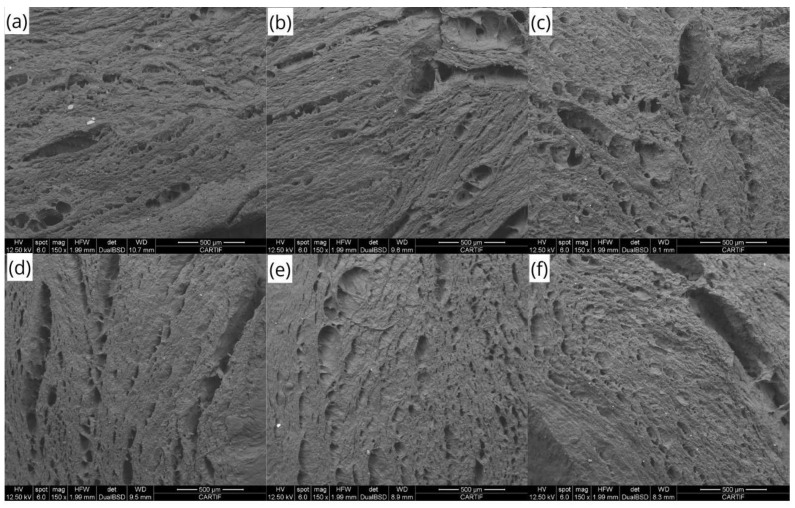
Microstructure of the high-moisture extrudates cut transversal to the flow direction under different temperatures (T) and screw speed (S) conditions. (**a**) LT-LS; (**b**) LT-MS; (**c**) LT-HS; (**d**) HT-LS; (**e**) HT-MS; (**f**) HT-HS. The scale bar in each image indicates 500 µm. LT, low temperature; HT, high temperature; LS, low speed; MS, medium speed; HS, high speed.

Scanning microscopy provided more detailed information on the protein network at the microscopic level (Figure 5). At the microscopic level, it was also observed that soy extrudates displayed anisotropy in the form of fibrous/layered structures, without clear differences between the different processing conditions.

From the visual appearance (Figure 4), darker meat analogs were also noted at high-temperature extrusion conditions, in agreement with colorimetric results. Thus, the extrusion parameters also influenced the color attributes. The color parameters of the protein powders and the extrudates are reported in Table 5. Both barrel temperature and screw speed showed a significant effect (*p* < 0.05), especially the former, on the lightness L* and the chromatic parameter b* (yellowness). However, only temperature had a significant effect on the a* values (redness). Thus, the total color change (Δ*E*) of the extrudates compared to that of the raw material (SPM) was especially influenced by the thermal energy applied. All the extrudates showed a decrease in L* and increase in b* and a* values compared to the SPM powder, resulting in darker and more reddish/yellowish products. Among the extrudates, L* values ranged between 60.84 and 64.25, a* values ranged between 2.87 and 4.69, and b* values ranged between 17.42 and 20.15. At the same barrel temperature, lowering the screw speed generally resulted in an increased chromatic value (b*) and decreased lightness (L*). At the same screw speed conditions, a higher barrel temperature resulted in increased redness/yellowness (a*, b*), but decreased lightness (L*). All in all, the total color difference, Δ*E*, increased with higher temperatures (120 to 140 °C) and with lower screw speeds (400 to 300 rpm) at LT conditions, probably associated with increased Maillard/caramelization reactions with higher product temperatures and longer resident times, respectively. Wang et al. [76] reported similar results for the effect of barrel temperature on the color parameters, showing a significant drop in lightness as the barrel temperature rose from 120 to 160 °C.

#### 3.4.2. Changes in Protein Secondary Structures upon Extrusion

**Figure 6:**
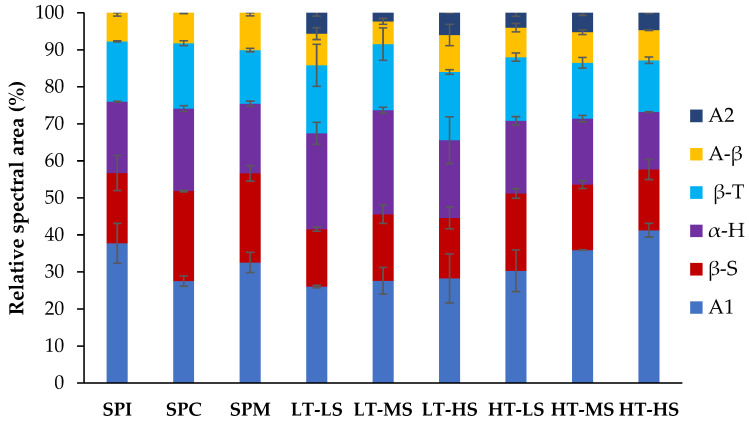
Relative FTIR spectral areas, expressed as percents of the total area, of the secondary structure bands of protein powders and high-moisture extruded meat analogs obtained via the Gaussian/Lorentzian deconvolution of the amide I band (between 1600 and 1700 cm^−1^). A1: aggregated structures; β-S: β-sheets; α-H: α-helix; β-T: β-turns; A-β: antiparallel β-sheets; A2: aggregated structures; SPI: soy protein isolate; SPC: soy protein concentrate; SPM: soy protein mix; LT: low temperature; HT: high temperature; LS: low speed; MS: medium speed; HS: high speed.

The changes in the secondary structures of the proteins after extrusion were studied using FTIR. The amide I region, C=O-stretching vibration band at 1600–1700 cm^−1^ was resolved to reveal the secondary structure components present in the protein powders and high-moisture extrudates. Examples of the deconvoluted FTIR spectra are included in the Supplementary Materials (Figure S1). The relative proportions of the secondary structures are shown in Figure 6. Before extrusion treatment, the SPM, the SPC:SPI mixture used for extrusion, denoted a secondary structure more similar to the SPC. the SPM was composed of bands associated with intermolecular protein-aggregated structures and the absorption of amino acid side chains (A1), β-sheets (βS), α-helices (α-H), β-turns (βT) and antiparallel β-sheets (A-β) as the main secondary structure components. These components were in agreement with other results in soy proteins [43,44]. In these protein powders, intermolecular aggregates A1 predominated, followed by β-sheets, while lower proportions of α-helices were visible, in agreement with Carbonaro et al. [43].

Upon extrusion, a significant reduction in the proportion of the band attributed to β-sheets structures, together with an increase of the A1 band, corresponding to intermolecular aggregates, was generally observed in the extruded meat analogs. Furthermore, a small proportion of a new band (~1690–1695 cm^−1^), related to intramolecular aggregates (A2), was also visible in all extrudates. Similar results were reported in soy concentrates after low-moisture extrusion [44]. The high temperature would have caused protein molecular unfolding and the formation of new protein structures or aggregates, including those of an intramolecular nature. Still, intermolecular aggregates were more abundant than intramolecular ones after extrusion treatment [44]. Protein molecular weight aggregation in soy protein isolates has been reported as a consequence of extrusion [77]. Regarding processing parameters, results denoted a higher presence of A1 aggregates at HT and HS extrusion conditions, with no clear differences for β-sheets or A2 aggregates among the different extruded meat analogs. Nonetheless, the sum of aggregated structures (A1 + A2) was higher at HT and HS conditions. Beck et al. [78] also reported that intermolecular aggregates in heat-treated pea protein isolates, which could be made of β-turn structures or antiparallel β-sheets, increased during heat treatment when shear force was applied. However, they also reported no clear trend in the proportion of intra-molecular aggregates with processing parameters. Meanwhile, Carbonaro et al. [43] also reported that legume proteins mainly possessed β-sheet and β-turn structures and lower proportions of α-helices before processing, with the loss of the β-sheet contribution in parallel with an increased formation of heat-induced A1 and/or A2 stable aggregates after autoclaving.

#### 3.4.3. Textural Properties of Extruded Meat Analogs

The textural attributes of the high-moisture extrudates obtained in the TPA analysis and cutting test are listed in Table 6. Regarding TPA analyses, hardness values ranged from 86 to 128 N, while smaller differences were observed for resilience, cohesiveness, and springiness, the latest not being significantly different among the extruded meat analogs. The process parameters, screw speed, and barrel temperature had an effect on the textural attributes, with higher hardness values found at lower processing temperatures (LT > HT) and screw speeds (LS > HS). The hardest extrudate was obtained at 120 °C (LT) and 300 rpm (LS) combined conditions. These results are consistent with those obtained by Lin et al. [28], showing that at a fixed moisture content, a higher cooking temperature resulted in softer and less chewy soy protein extrudates, partially related to an increase in protein solubilization. Higher barrel temperatures (140 °C) may result in lower melt viscosities due to the higher molecular mobility of the SPM components [17]. Thus, higher extrusion temperatures and resident times at these high temperatures could have fostered polysaccharides degradation, protein denaturation, and/or the rupture and reformation of new aggregates, in line with the different viscosity results observed when heat-processing the SPM at different maximum temperatures (Figure 3) and with the changes in the protein secondary structure of the extrudates (Figure 6). In fact, the motor toque and melt pressure at the extruder exit (Table 2) were lower at HT (140 °C) and HS (400 rpm) conditions, also supporting the lower hardness of these extruded products, as less viscous melted extruded materials would result in lower melt pressure conditions [17]. For resilience and cohesiveness, small changes were noticed, and only low screw speed conditions seemed to result in more resilient and cohesive extrudates, which may be related to the longer resident times of the melted extruded material inside the heated barrels. Zahari et al. [72] also found that the increase in hardness was generally reflected with an increase in the resilience of the high-moisture extrudates.

The cutting force or cutting strength, defined as the energy required to cut the sample, can be used to analyze the extent of fiber formation in the extrudates if cutting is performed parallel (L) and perpendicular (T) to the flow direction of the extrudates in the cooling die. The fibrous degree is also referred to as the anisotropic index (FL/FT) [27]. Equal values of FL = FT indicate a uniform isotropic texture, while FT > FL or FL > FT values indicate the predominance of longitudinal or transverse fibers, respectively [79]. The effects of screw speed and temperature on the cutting strength and degree of texturization are reported in Table 6 In all cases, transversal cutting force was higher than the longitudinal one, denoting the presence of anisotropic structures (FT > FL), with fibers oriented longitudinal to the flow direction. This would agree with the macroscopic observations of fiber directions in Figure 4. Values obtained for the cutting force lengthwise were between 2.02 and 5.23 N, and for the transverse force were between 2.46 and 7.23 N. The degree of texturization (FL/FT) was lower for low-temperature conditions (120 °C), as values closer to 1 were obtained, and hence, were closer to more isotropic structures (FL = FT). However, no clear effect for screw speed conditions was observed, which was also in agreement with visual observations. According to Bouvier and Campanella [80], an increase in melt viscosity results in a flatter velocity profile in the cooling die channel, while lowering the viscosity (i.e., by increasing cooking temperature) has the opposite effect, resulting in an extended velocity profile, facilitating the development of flow lines within the melt that, upon cooling, results in elongated fiber formation. This would be in line with the lower pressure (indicative of a lower melt viscosity) observed at HT conditions, when more fibrousness was observed.

#### 3.4.4. Reduction of Trypsin Inhibitors upon Extrusion

**Figure 7:**
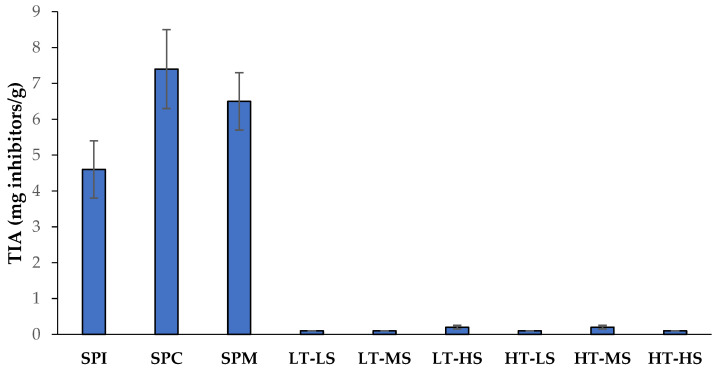
Trypsin inhibitors (TIA, mg inhibitors/g solids of product, in a wet basis) for protein powders and extruded meat analogs produced at different temperatures and screw speed conditions. SPI: soy protein isolate; SPC: soy protein concentrate; SPM: soy protein mix; LT: low temperature; HT: high temperature; LS: low speed; MS: medium speed; HS: high speed.

Figure 7 shows the trypsin inhibitor (TI) content of soy protein powders and high-moisture extruded meat analogs obtained at different screw speed and temperature conditions. Regarding protein powders, SPC showed significantly higher trypsin inhibitor contents than SPI, with values of 7.4 and 4.6 mg/g (14.1 and 8.7 U/mg), respectively. Likewise, previous research reported values in the range of 7.3–5.4 mg/g for an SPC and 3.6–7.1 mg/g for an SPI [81]. Similarly, other studies have reported a 70–90% reduction in trypsin inhibitor concentrations or activity during the concentration and purification of protein from soybean meals [22,23].

All high-moisture extruded products showed a decrease in the amount of trypsin inhibitors as compared to the SPM (6.5 to 0.1–0.2 mg/g, w.b.). This effect was not only the result of the dilution of the SPM in the extruded product as a consequence of the high-water content used for extrusion (~70%), but was also the result of the high thermal instability of trypsin inhibitors [82,83]. Thus, considering SPM concentration in the extruded meat analogs (i.e., correcting to their solid contents), trypsin inhibitors were reduced by more than 90% after extrusion treatments. The combination of high temperatures, pressure, and mechanical forces during the extrusion process leads to certain non-reversible structural changes in trypsin inhibitor conformations, leading to their denaturation, and the loss of inhibitory enzymatic activity, consequently improving the nutritional quality of the final products [82,84]. Likewise, other authors have reported ~90% reductions in trypsin inhibitor activities after the low-moisture extrusion of breadfruit–corn–soy composites, with no significant effect of screw speed on the TIA [25]. Conversely, in another study at low-moisture conditions, Petres and Czukor [26] reported the destruction of trypsin inhibitors in soybean flour as a function of temperature, screw speed (residence time), and moisture level. However, in our study, performed in high-moisture conditions, it seems that the processing variables selected were severe enough to achieve a high degree of removal of the trypsin inhibitors, not finding significant differences among the studied parameters. In line with these results, other authors have reported that higher temperatures are needed to inactivate TIs under low-moisture conditions, being that higher moisture conditions are recommended to effectively remove TIs, especially if low extrusion temperatures are applied (<130 °C) [24,85].

## 4. Conclusions

To better understand the effects of extrusion processing parameters on the textural and nutritional quality of high-moisture extruded meat analogs, this study examined the micro- and macrostructure, textural attributes, color, protein secondary structure, and trypsin inhibitors of soy protein-based extrudates. The high-moisture extrudates were produced at two different barrel temperature profiles and three screw speeds from a well-characterized mixture (SPM) of a soy protein isolate (SPI) and concentrate (SPC). SPC presented a lower extent of protein denaturation and aggregation (observed in DSC and FTIR), a lower least gelation concentration, and higher total essential amino acid contents compared to the SPI. Nonetheless, the SPI exhibited a higher gel strength after gelation in a heating–cooling cycle, and enhanced foaming and emulsifying properties, which may be related to its higher protein purity (>90%). Therefore, the combined SPM used for extrusion exhibited enhanced functional properties compared to those of SPC and SPI, respectively.

During high-moisture extrusion of soy protein, a high level of both visual and instrumental anisotropy was observed in all extruded meat analogs, especially when higher extrusion temperatures were selected. The higher level of anisotropy at higher extrusion temperatures could be related to a greater level of denaturation of the 11S or the molecular disassembling of protein aggregates in the raw material, which could then realign more efficiently via the directional shear force in the cooling die, creating more fibrous structures. These fibers would presumably be stabilized by increased protein aggregation, as suggested by the more effective reassociation visible in the FTIR, by increasing the level of inter- and intramolecular aggregates (A1 and A2 structures) reformed in these extrudates. It is noteworthy that both process parameters, screw speed and temperature, greatly influenced both the textural attributes, with softness increasing at higher temperatures and higher speeds, and the color attributes, with darker extrudates at higher temperatures and lower screw speeds (i.e., longer resident times). Regarding the antinutritional factors, trypsin inhibitors were significantly reduced after extrusion (>90% reduction), without any clear effect of the temperature or speed parameters, which may be related to the thermolabile nature of this antinutrient and the high temperatures used during extrusion (>120 °C). Findings indicated that the quality of the extrudates was heavily influenced by the selected extrusion parameters, highlighting the importance of carefully selecting extrusion conditions to achieve meat analogs with optimal textural and nutritional characteristics. Thus, higher temperatures are recommended to obtain a greater fibrousness for soy protein extruded products, while the effect of screw speed on fibrousness was less noticeable, although differences in resident times (screw speeds) influenced the hardness and color attributes of the product.

## Figures and Tables

**Table 1:** Pasting profiles obtained at 95, 120, and 140 °C maximum temperatures (adapted from [42]).

Parameter	Value	Time (min:s)		
		95 °C	120 °C	140 °C
Initial Temperature (°C)	50	00:00	00:00	00:00
Speed (rpm)	960	00:00	00:00	00:00
Speed (rpm)	160	00:10	00:10	00:10
Temperature (°C)	50	01:00	01:00	01:00
Temperature (°C)	Hold *	08:30	12:40	16:00
Temperature (°C)	Hold *	13:30	17:40	21:00
Temperature (°C)	50	21:00	29:10	36:00
End Temperature (°C)	50	23:00	31:10	38:00

* Hold period for 5 min at maximum temperature of 95, 120, or 140 °C, respectively, for each pasting cycle.

**Table 2:** The operational extrusion conditions for screw speed and temperature profiles, specific mechanical energy (SME) and product melt temperature and pressure resulting from the extrusion experiments.

Barrel Segment Temperatures (°C)						Screw Speed (rpm)	Motor Torque (%)	Melt Temperature (°C)	Melt Pressure (bar)	SME (Wh/Kg)	Extrusion Conditions
Z1	Z2	Z3	Z4	Z5	Z6						
30	50	75	105	115	120	300	11.2	115	18.0	41.1	LT-LS
						350	12.3	117	20.4	52.6	LT-MS
						400	9.2	115	14.3	44.9	LT-HS
30	70	95	115	125	140	300	10.4	131	16.5	38.1	HT-LS
						350	9.8	130	15.5	41.9	HT-MS
						400	7.9	130	11.6	38.7	HT-HS

SME: specific mechanical energy; LT: low temperature; HT: high temperature; LS: low speed; MS: medium speed; HS: high speed.

**Table 3:** The proximate composition and physicochemical properties of soy protein ingredients.

	SPI	SPC	SPM
*Proximate composition*			
*Moisture (g/100 g, w.b.)*	*7.6 ± 0.2 b*	*6.1 ± 0.1 a*	6.3 ± 0.2 a
*Protein (g/100 g d.b.)*	*95.4 ± 1.1 c*	*73.9 ± 0.1 a*	76.5 ± 0.1 b
*Crude Fiber (g/100 g d.b.)*	*0.8 ± 0.0 a*	*3.5 ± 0.1 c*	3.1 ± 0.1 b
*Carbohydrates (g/100 g d.b.)*	*0.2 ± 0.0 a*	*11.7 ± 0.1 c*	10.3 ± 0.1 b
*Lipids (g/100 g d.b.)*	*0.4 ± 0.1 a*	*0.6 ± 0.1 a*	0.6 ± 0.1 a
*Ash (g/100 g, d.b.)*	*4.0 ± 0.1 a*	*4.4 ± 0.2 a*	4.3 ± 0.2 a
*Physicochemical properties*			
T_d1_ (°C)	nd	86.4 ± 0.7 a	86.1 ± 1.9 a
T_d2_ (°C)	nd	116.6 ± 0.1 a	117.4 ± 0.6 a
ΔH_1_ (J/g _protein_)	nd	1.8 ± 0.6 a	1.4 ± 0.1 a
ΔH_2_ (J/g _protein_)	nd	3.4 ± 0.1 b	3.2 ± 0.1 a
WBC (g/g)	4.7 ± 0.1 a	5.0 ± 0.1 b	5.0 ± 0.0 b
OBC (mL/g)	3.4 ± 0.1 a	3.0 ± 0.2 a	3.0 ± 0.1 a
LGC (%)	14 ± 0 c	12 ± 0 a	13 ± 0 b
FC (%)	43.1 ± 2.0 a	50.0 ± 0.0 b	49.2 ± 0.2 b
FS (%)	79.2 ± 5.9 b	62.7 ± 0.2 a	64.7 ± 0.9 a
EAI (m^2^/g)	99.5 ± 0.0 b	94.5 ± 0.3 a	95.1 ± 0.2 a
ESI (min)	1350 ± 0.0 c	80.2 ± 0.2 a	555.0 ± 0.2 b

Mean ± standard deviation followed by different letters within each row indicate significant differences (*p* < 0.05). SPI: soy protein isolate; SPC: soy protein concentrate; SPM: soy protein mix; T_d_: denaturation temperature at peak maximums of peaks 1 and 2; ΔH: the enthalpy of protein denaturation of peaks 1 and 2; nd: not detected; WBC: water-binding capacity; OBC: oil-binding capacity; LGC: least gelation concentration; FC: foaming capacity; FS: foaming stability, EAI: emulsion activity index, ESI: emulsion stability index.

**Table 5:** Results of the CIE of the L*, a*, and b* color parameters for protein powders and high-moisture extruded meat analogs and the Δ*E* of extrudates as compared to the soy protein mixture.

	L*	a*	b*	Δ*E*
SPI	86.07 ± 0.06 e	−0.27 ± 0.01 a	16.33 ± 0.09 b	-
SPC	88.05 ± 0.00 g	−0.55 ± 0.01 a	14.58 ± 0.02 a	-
SPM	87.38 ± 0.05 f	0.10 ± 0.01 a	14.68 ± 0.08 a	-
LT-LS	64.25 ± 0.23 c	3.22 ± 0.03 b	18.85 ± 0.19 de	23.71 ± 0.25 b
LT-MS	64.55 ± 0.22 c	3.29 ± 0.07 b	18.59 ± 0.05 d	23.38 ± 0.28 b
LT-HS	66.08 ± 0.17 d	2.87 ± 0.01 b	17.42 ± 0.25 c	21.65 ± 0.19 a
HT-LS	60.84 ± 0.25 a	4.31 ± 0.25 c	19.66 ± 0.24 ef	27.33 ± 0.22 d
HT-MS	61.83 ± 0.32 b	4.69 ± 0.47 c	20.15 ± 0.93 f	26.53 ± 0.51 cd
HT-HS	61.74 ± 0.4 b	4.24 ± 0.88 c	18.95 ± 0.79 de	26.32 ± 0.72 c

Mean ± standard deviation followed by different letters within each column indicate significant differences (*p* < 0.05). SPI: soy protein isolate; SPC: soy protein concentrate; SPM: soy protein mix. Δ*E*: total color difference of the high-moisture extrudates compared to their unextruded protein powder (SPM). LT: low temperature; HT: high temperature; LS: low speed; MS: medium speed; HS: high speed.

**Table 6:** Textural properties of the high-moisture extruded meat analogs obtained at different screw speeds and barrel temperatures.

Conditions	Hardness	Resilience	Cohesiveness	Springiness	Cutting Force Longitudinal (FL)	Cutting ForceTransverse (FT)	FL/FT
	N	%	%	%	N	N	
LT-LS	127.62 ± 8.37 c	0.41 ± 0.03 bc	0.81 ± 0.02 ab	0.78 ± 0.02 a	3.18 ± 0.15 b	3.73 ± 0.25 b	0.85 ± 0.05 c
LT-MS	113.60 ± 3.52 b	0.40 ± 0.03 ab	0.80 ± 0.02 ab	0.78 ± 0.04 a	2.95 ± 0.31 b	3.65 ± 0.22 b	0.81 ± 0.07 c
LT-HS	111.48 ± 1.35 b	0.35 ± 0.04 a	0.78 ± 0.04 a	0.74 ± 0.04 a	2.02 ± 0.08 a	2.46 ± 0.09 a	0.82 ± 0.05 c
HT-LS	109.06 ± 6.97 b	0.47 ± 0.07 c	0.83 ± 0.04 b	0.78 ± 0.04 a	5.23 ± 0.27 d	7.23 ± 0.44 e	0.73 ± 0.05 b
HT-MS	89.25 ± 4.41 a	0.39 ± 0.06 ab	0.76 ± 0.05 a	0.78 ± 0.07 a	4.05 ± 0.16 c	6.31 ± 0.27 d	0.64 ± 0.04 a
HT-HS	86.16 ± 4.90 a	0.37 ± 0.06 ab	0.76 ± 0.05 a	0.78 ± 0.09 a	4.01 ± 0.15 c	5.62 ± 0.24 c	0.72 ± 0.03 b

Mean ± standard deviation followed by different letters within each column indicate significant differences (*p* < 0.05). FL: longitudinal; FT: transverse; LT: low temperature; HT: high temperature; LS: low speed; MS: medium speed; HS: high speed.

## Data Availability

Data are contained within the article or in the Supplementary Materials. Further inquiries can be directed to the corresponding author.

## Supplementary Materials

The following supporting information can be downloaded at: https://www.mdpi.com/article/10.3390/foods13111748/s1, Table S1: Peak maximum viscosity and final viscosity obtained from the pasting profiles of SPI, SPC and SPM at 95 °C, and for SPM at 120 °C and 140 °C; Figure S1: FTIR-ATR spectra of the soy protein mix and extruded soy.
