# Supplementary material for: Optimizing Screw Speed and Barrel Temperature for Textural and Nutritional Improvement of Soy-Based High-Moisture Extrudates
Source: Foods. 2024 Jun 2;13(11):1748. doi: 10.3390/foods13111748 (PMC11171804; doi:10.3390/foods13111748)
Supplement: Supplementary file 1 [file foods-13-01748-s001.zip › foods-3025684-supplementary.pdf]

Supplementary material

Optimizing Screw Speed and Barrel Temperature for Textural and Nutritional Improvement of Soy-Based High-Moisture Extrudates

Gabriela Ribeiro, María-Ysabel Piñero, Florencia Parle, Belén Blanco, Laura Román

Table S1. Peak maximum viscosity and final viscosity obtained from the pasting profiles of SPI, SPC and SPM at 95 °C, and for SPM at 120 °C and 140 °C.

Sample	Cold peak Viscosity (mPa·s)	Final Viscosity (mPa·s)
**SPI - 95 °C**	2833.5±43.9b	3334.5±24.2c
**SPC - 95 °C**	1567.0±8.5a	462.5±10.6a
**SPM - 95 °C**	2315.0±41.4ab	860.5±14.1ab
**SPM - 120 °C**	2372.5±36.3ab	1282.0±30.9b
**SPM - 140 °C**	2511.0±58.6b	1249.0±25.8b

Mean ± Standard deviation followed by different letters within each row indicate significant differences (P<0.05). SPI: soy protein isolate; SPC: soy protein concentrate, SPM: soy protein mixture.

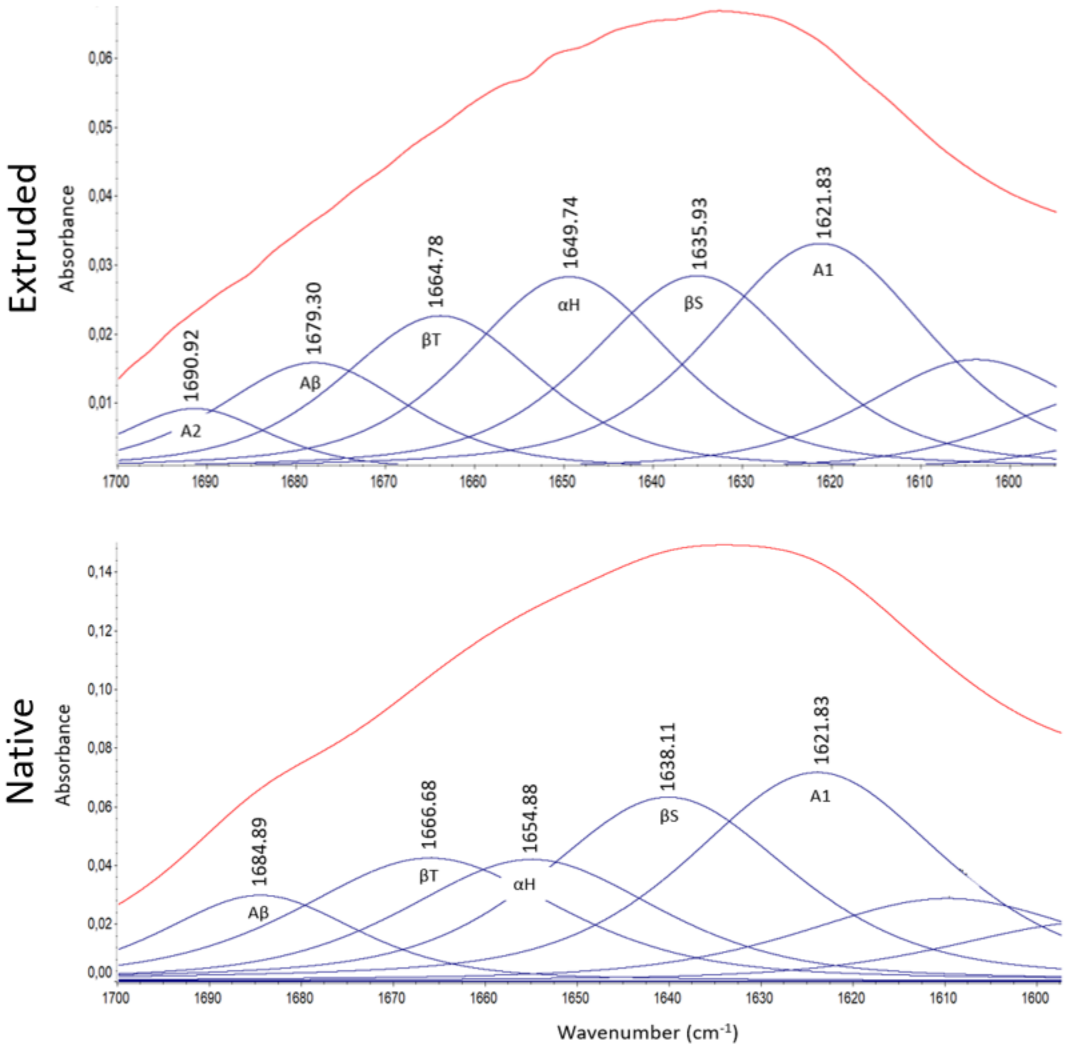

Figure S1. FTIR-ATR spectra of soy protein mixture (SPM) and extruded SPM. Native protein SPM and extruded HT-MS have been considered. In SPM peaks correspond to: A1 (1621.83 cm^{-1}); β-sheet (1638.11 cm^{-1}); α-Helix (1654.88 cm^{-1}); β-turns (1666.68 cm^{-1}); antiparallel β-sheet (1684.89 cm^{-1}); for extruded HT-MS peaks correspond to: A1 (1621.83 cm^{-1}); β-sheet (1635.93 cm^{-1}); α-Helix (1649.74 cm^{-1}); β-turns (1664.78 cm^{-1}); antiparallel β-sheet (1679.30 cm^{-1}); A2 (1690.92 cm^{-1}).
